# Growth performance, carcass traits, and histological changes of goats supplemented with different sources of phytochemicals

**DOI:** 10.1038/s41598-025-07306-w

**Published:** 2025-06-23

**Authors:** Alaa Emara Rabee, Osama Raef, Ahmed M. Sallam, Eman Ali Elwakeel, Rasha S. Mohammed, Ibrahim S. Abd El‐Hamid, Mebarek Lamara, Adel M. Abdel-Wahed, Mohamed Ali Radwan, Ibrahim M. Khattab, Moustafa Mohamed M. Ghandour

**Affiliations:** 1 Animal and Poultry Nutrition Department, Desert Research Center, Ministry of Agriculture and Land Reclamation, Cairo, Egypt; 2 Animal and Poultry Breeding Department, Desert Research Center, Ministry of Agriculture and Land Reclamation, Cairo, Egypt; 3 Animal and Fish Production Department, Faculty of Agriculture, Alexandria University, Alexandria, Egypt; 4 Animal and Poultry Health Department, Desert Research Center, Ministry of Agriculture and Land Reclamation, Cairo, Egypt; 5 Animal and Poultry Physiology Department, Desert Research Center, Ministry of Agriculture and Land Reclamation, Cairo, Egypt; 6 Forest Research Institute, University of Quebec in Abitibi-Temiscamingue, Rouyn-Noranda, Canada; 7 Animal Production Department, Faculty of Agriculture, Cairo University, Cairo, Egypt; 8 Animal and Fish Production Department, Faculty of Desert and Environmental Agriculture, Matrouh University, Matrouh, Egypt

**Keywords:** Shami goats, Phytochemicals, Tannin, Herbal mixture, Performance, Immunity, Carcass traits, Physiology, Zoology, Planetary science

## Abstract

Phytogenic feed additives are increasingly used to improve animal health and productivity. This study compared the effect of supplementation with tannin to an herbal mixture consisting of ginger, garlic, artemisia, and turmeric on the performance, intestinal parasites, blood metabolites, carcass characteristics, and histology of muscles and intestine of goats. Twenty-seven Shami male goats were assigned to three treatments (n = 9): non-supplemented goats fed a control diet (CC); goats supplemented with 10g /animal/day of quebracho tannins as a source of condensed tannin (TT); and goats supplemented with 10g/animal/day of an herbal mixture (HM). All the animals received a basal diet consisted of concentrate feed mixture and alfalfa hay. The supplementation improved growth performance, nutrients digestibility, and serum immunoglobulins concentration (*P* < 0.05). The supplementation decreased fecal parasite counts, blood cholesterol, and glutamic-pyruvic transaminase (GPT) enzyme and improved blood glucose (*P* < 0.05). The supplementation decreased renal and meat fat, and group HM revealed higher polyunsaturated fatty acids and α-Linolenic acid in meat (*P* < 0.05). Tannin supplementation (TT group) negatively affected the histology of muscles and intestines. The results provide evidence for the beneficial use of an herbal mixture in the diet to improve animal performance, health status, and meat quality in goats.

## Introduction

Phytochemicals are emerging feed additives to promote animal health, feed efficiency, and the quality of animal products, as well as their safe applications^1^. Common plants that contain phytochemicals include ginger (*Zingiber officinale* Roscoe), garlic (*Allium sativum* L*.*), artemisia (*Artemisia vulgaris* L*.*), and turmeric (*Curcuma longa* L*.*). Ginger has antioxidant, anti-inflammatory, immunomodulatory, and antimicrobial properties due to its content of several substances such as gingerol and gingerdione^2^. Garlic contains several organosulfur compounds, including allicin, diallyl sulfide, γ-glutamylcysteine, and S-allyl cysteine, which trigger its effect as a growth promoter, immune-modulator, anti-inflammatory, anti-oxidative stress, anti-hyperlipidemia, and anti-parasite^3^. Artemisia contains artemisinin, which has anticoccidial and antiparasitic activities^4^. Turmeric contains oils, curcumin, and turmerones, which have antimicrobial and antioxidant activities^5^.

Phenolic compounds (tannins, saponins, and flavonoids) are the phytogenic substances found in ginger, garlic, artemisia, and turmeric^1–4^. Therefore, these herbal plants were combined in several phytogenic mixtures to obtain their synergistic effect to promote animal performance^1,6,7^. The study of Redoy et al.^1^ reported that sheep supplemented with herbal mixtures demonstrated higher weight gain, immunoglobulins, and lower carcass caul and pelvic fat, and meat-saturated fatty acids. Similarly, Razo Ortiz et al.^6^ supplemented the lambs with polyherbal feed additives and noticed that the supplementation increased daily gain and rumen total volatile fatty acid (VFA). Additionally, Szulc et al.^8^ showed that herbal mixture supplementation improved the total VFA and dry matter (DM) digestibility in nematode-infected lambs. On the other hand, extracted phytochemical compounds were used separately to promote the feed efficiency of farm animals. For example, Min et al.^9^ supplemented goats with tannin‐rich peanut skin and observed increments in daily gain, and some carcass characteristics such as empty weight. Furthermore, Orzuna-Orzuna et al.^7^ reported that tannin supplementation in sheep did not affect feed intake or meat chemical composition but increased daily gain and total antioxidant. Supplementing lactating buffalos with flavonoids improved the production of microbial protein and VFAs^10^. Limited information is available on comparing the effects of phytogenic mixtures to single extracted phytocompounds, such as tannins, on animal performance. The Shami goat breed is one of the main goat breeds in Mediterranean countries because it plays an important role in food security in arid and semiarid regions as it produces milk and meat under harsh conditions^11^. However, goat production in arid regions is challenged by the shortages in food resources and health problems^12,13^. Therefore, using feed additives such as phytochemicals could improve animal performance and health, and increase the quality of animal products. The effect of phytochemicals on animal performance was studied on several animal species, but limited information is available on the effect of phytochemicals on the performance of Shami goats. Moreover, it was hypothesized that the herbal mixture that contains a mixture of phytogenic compounds might show a synergistic activity and a better effect on animal performance than extracted separate types of phytochemicals (tannin, flavonoids, and saponins), yet no information is available in this regard. Therefore, the goal of this study was to investigate the effect of condensed tannin and herbal mixtures on the performance, digestibility, blood metabolites and immunity, intestinal parasites, carcass characteristics, and meat quality of Shami goats.

## Materials and methods

### Ethics

All the methods in this experiment, including euthanasia of the animals, were performed in accordance with the guidelines and regulations of the Animal Care and Use Committee in the Department of Animal and Poultry Production, Desert Research Center, Egypt. Furthermore, the Alexandria University Research Ethics Review Committee, Faculty of Agriculture, University of Alexandria, Egypt, approved the experiment (Reference: Alex. Agri. No: 082305305). All methods and protocols in this study comply with the ARRIVE 2.0 guidelines and the EU standards for the protection of animals. The origin of all tools, kits, and devices is presented in Supplementary File 1.

### Animals, diets, and experimental design

This study was conducted at Maryout Research Station, Desert Research Center, Alexandria, Egypt. This study is a part of a project that includes another published study^14^ that investigated the effect of the same current treatments on rumen fermentation and microbiota. In this study, twenty-seven male Shami goats (11 months of age, 25.60 ± 1.25 kg body weight) were selected randomly for this 100-day study. The goats used in this study belonged to the goats’ herd of Maryout Research Station, Desert Research Center, Alexandria, Egypt, and the animals were used in the experiment using the required consent from the administration of Maryout Research Station and the Animal and Poultry Production Division. Goats were housed in shaded pens, and drinking water was offered twice a day. Animals received an experimental diet consisting of 70% concentrate feed mixture and 30% Alfalfa hay (*Medicago sativa* L*.*) to meet the growth requirements. The concentrate feed mixture consisted of 60% corn, 12% soybean meal, 12% wheat bran, 8% barley grain, 5% cotton meal, 1.3% limestone, 0.5% salt, 0.3% sodium bicarbonate, 0.7% premix, and 0.2% antitoxins. The proximate chemical composition for alfalfa hay on the dry matter (DM) basis was: 842.4 g/Kg organic matter (OM), 155 g/Kg crude protein (CP), and 584.5 g/Kg neutral detergent fiber (NDF). Moreover, the proximate chemical composition for concentrates feed mixture on the DM basis was: 911 g/Kg OM, 165 g/Kg CP, and 291.5 g/Kg NDF. Animals were divided randomly to three experimental groups (n = 9): group (CC) was the control group and received the basal diets without additives, group (TT) received the basal diet supplemented with quebracho tannins as a source of condensed tannins (10 g/head/day), and group (HM) received the basal diet supplemented with an herbal mixture (10 g/head/day). The herbal mixture consisted of ginger (*Zingiber officinale*), garlic (*Allium sativum*), artemisia (*Artemisia vulgaris*), and turmeric (*Curcuma longa*), which were obtained from the commercial market were mixed in equal quantities (1:1:1:1). The condensed tannins and herbal mixture were offered gradually and supplied to the animals at 1% of DM feed intake^14^, and to provide 2.5 g/kg DM feed intake from every herbal ingredient according to the recommendations of previous studies^4,15,16^. All experimental additives were mixed with the concentrate feed mixture to confirm full intake. Orts were weighed, and feed intake was recorded daily. Diet and orts were sampled weekly and dried in a forced-air oven at 65 °C for 48 h. The animals were weighed at the beginning and the end of the experiment.

### Digestibility trail

At the end of the experiment, the nutrients’digestibility trial was conducted. Goats were kept in the metabolic cages for seven days for adaptation, followed by seven days for urine and fecal collection. Animals were weighed at the beginning and end of the trial. During the collection period, the offered and refused feeds were recorded, and representative samples were collected daily. Daily feces output was collected and mixed thoroughly, and a 10% sub-sample of each animal was collected. Subsamples of feces of offered and refused feeds were pooled into individual samples for each animal for the seven-day collection period and dried at 65 °C for 48 h. Dried samples were ground and stored for further analysis. Urine was collected every day in jars, acidified using 100 ml of 4 N sulphuric acid (H_2_SO_4_), quantified, and a 10% sub-sample of each animal was collected for nitrogen determination. During the collection period, drinking water intake was recorded. Daily water excretion in feces and urine was measured. The digestibility of the nutrients was determined according to the method described by McDonald et al.^17^.

### Blood sampling

Blood samples were collected from the goats at the end of the experimental period. The samples were collected before morning feeding from the jugular vein, and blood serum was separated by centrifuging at 10,000 × g for 5 min. The serum samples were then stored at −20 °C for further analysis.

### Animal euthanasia, carcass traits, and meat quality

At the end of the experiment, the animals were fasted for 12 h with free access to water. The animals were brought to Maryout Research Station’s slaughterhouse, Alexandria, Egypt, where the slaughter was conducted. Animals were euthanized manually by an experienced slaughterer by cutting all the arteries and veins at the throat area in the neck with a very sharp knife, minimizing any injury or stress to the animal, and no electrical stimulation, stunning, or chemical treatments were used. Furthermore, the animals were not anaesthetized or unconscious during the slaughtering. The death of the animals was ensured before further processing and sampling. After bleeding, skinning, and evisceration, the hot carcass weight was recorded to calculate the dressing percentage according to Zayed et al.^18^. Carcass cuts in terms of neck, shoulders, rack, flank, loin, legs, and tail were weighted. Also, edible organs like the liver, heart, and kidneys were weighed. The best ribs (9, 10, and 11 ribs) were separated and dissected into meat, fat, and bone, which were weighted separately to determine the proportions of bone, meat, and fat and to quantify the fat in meat. The nutritive value of the best ribs meat was analyzed by a meat analyzer. Meat color was measured in the best ribs muscle using a chroma meter. A total of three spectral readings were taken for each sample at different positions on the cuts. Where lightness (L*) (dark 0 to 100 light), and redness (a*) values range from reddish (+ value) to greenish (- value). The yellowness (b*) values: yellowish (+ value) to bluish (- value). Also, Chroma and Hue were measured.

### Chemical composition

Dried feed, and fecal samples were ground and analyzed according to AOAC^19^ to measure dry matter (DM, method 930.15), crude protein (CP, method 954.01), and ether extract (EE, method 920.39). Neutral detergent fiber (NDF) was measured using ANKOM Technology according to the method of Van Soest et al.^20^. Urine nitrogen was analyzed according to AOAC^19^. Total phenolics, total flavonoids, total tannins, and total saponins were determined in the herbal mixture. Total flavonoids were extracted using petroleum ether and 95% ethanol and determined according to the method of Karawaya and Aboutabl^21^. Total phenols and total tannins were determined according to the method of Makkar^22^. Total saponins were determined using 70% ethanol extraction, according to the method of Kurkin and Ryazanova^23^.

### Serum metabolites analysis

The analyses of serum glucose (GLU, mg/dL), cholesterol, (CHO, mg/dL), triglycerides (TG, mg/dL), creatinine (CRA, mg/dL), albumin (ALB, g/dL), total protein (TP, g/dL), urea (UREA, mg/dL), glutamic pyruvic transaminase (GPT, IU/L), glutamic oxaloacetic transaminase (GOT, IU/L), were determined using commercial kits according to the manufacturer’s recommendations. Furthermore, Immunoglobulin A, Immunoglobulin G, and Immunoglobulin M were measured using Enzyme-Linked Immunosorbent Assay (ELISA).

### Fecal parasite analysis

At the end of the experiment, fresh fecal samples were collected from the rectums of all animals and stored in a sterile 50 mL tube containing 10% formalin solution at 4 °C for further examination. Microscopic examination was applied to detect the fecal parasites according to the protocols of Garcia^24^, and the presence of *Giardia lamblia* was confirmed using the formol-ether concentration technique.

### Fatty acid composition in meat

The intramuscular fatty acids present in the LD muscles were extracted using the method outlined by Folch et al.^25^. The fatty acid composition was analyzed using Trac 1300 gas chromatography. Long-chain fatty acids were identified using a TG-5MS Zebron capillary column and helium as the carrier gas at a flow rate of 1 mL/min and split the ratio of 1:20.

### Histomorphological examination

Autopsy samples were collected from the intestine and muscle of slaughtered goats to conduct the histological examination. Samples were collected and fixed in a 10% neutral buffered formalin solution, then washed, dehydrated in ethyl alcohol of different grades, cleared in methyl benzoate, and embedded in paraffin wax. Blocks were processed using standard procedures. Sections (5-μm thick) were stained with hematoxylin and eosin and examined microscopically^26^.

### Statistical analysis

The effect of supplementation on the differences in feed intake, growth performance, digestibility, blood metabolites, and the count of intestinal parasites was tested using aone-way ANOVA in IBM SPSS software v. 20.0^27^ using the Duncan test at *P* < 0.05. The effect of sample size was determined using the “effect of size” option in the univariate feature in SPSS based on partial Eta squared, which was insignificant (> 0.05).

## Results

### Phytochemical compounds in the herbal mixture (HM)

The herbal mixture that was used in group HM consisted of ginger, garlic, artemisia, and turmeric; this mixture contained total phenolics 60 mg/100 g, total flavonoids 17.63 mg/100 g, total saponins 48.8 mg/100 g, and total tannins 49 mg/100 g.

### Feed intake and digestibility of nutrients

Feed intake of DM, OM, CP, EE, and NDF were similar between the experimental groups (Table 1), and water followed the same trend. The digestibility of DM, OM, and NDF were improved by dietary supplementation whenever group HM showed the highest apparent digestibility (Table 1) and nitrogen balance followed the same trend (*P* < 0.05).

**Table 1:** Nutrient digestibility, nitrogen balance, feed intake, parasite counts, and growth performance of goats supplemented with tannin (TT) and herbal mixture (HM).

	Treatments						P-value
	CC		TT		HM		
	Mean	SE	Mean	SE	Mean	SE	
**Digestibility, g/kg DM**							
Dry matter	701.80^a^	22.11	734.00^ab^	11.89	779.70^b^	11.30	0.02
Organic matter	721.10^a^	19.45	753.70^ab^	10.40	796.79^b^	10.00	0.013
Crude protein	764.60	15.23	758.50	11.40	800.20	13.17	0.11
Ether extract	800.40	10.63	821.90	14.13	827.50	6.94	0.23
Neutral detergent fiber	538.90^a^	27.90	571.90^a^	24.70	693.00^b^	15.90	0.003
Nitrogen balance, g/d	12.70^a^	0.72	11.21^a^	0.68	16.22^b^	0.34	0.001
**Feed Intake, g/kg**^0.75^							
Dry matter	71.07	1.60	69.60	1.18	69.70	1.17	0.80
Organic matter	63.05	1.47	62.12	1.00	62.09	1.02	0.81
Crude protein	13.15	0.30	12.96	0.20	12.95	12.95	0.81
Ether Extract	1.85	0.04	1.83	0.03	1.83	0.024	0.85
Neutral detergent fiber intake	25.97	0.82	25.26	0.49	25.38	0.66	0.74
Water Intake, ml/kg ^0.75^	220.87	14.94	250.8	25.46	233.60	27.44	0.67
**Parasites, cell/each field**							
Giardia Lamblia	7.5^b^	0.94	3.5^a^	0.56	2^a^	0.65	0.0001
Strongyloides	3.5^b^	0.56	1.75^a^	0.16	1.25^a^	0.16	0.0001
**Growth performance**							
Initial body weight, kg	28	1.08	26	3.57	25.6	1.29	0.77
Final body weight, kg	36.36	0.45	35.11	3.15	35.93	1.00	0.78
Daily gain, g/d	85.61^a^	7.50	85.18^a^	7.88	111.28^b^	3.88	0.037

^a,b,c^: Means in the same row with different superscripts differ significantly (*P* < 0.05).

### Prevalence of gastrointestinal parasites

The predominant protozoa was *Giardia lamblia*, which declined significantly (P < 0.05) in supplemented groups (TT and HM) compared with the control group (CC) (Table 1). Moreover, the predominant nematode species was *Strongyloides,* which was declined in the group TT and the HM group that was supplemented by the herbal mixture (Table 1).

### Growth performance

Animals in this study demonstrated similar initial body weight (Table 1). Average daily gain (ADG) (g/d) was higher (*P* < 0.05) in group HM compared with group CC and TT (Table 1).

### Blood biochemical and immunity profiles

Blood serum biochemical and immunoglobulins parameters are presented in Table 2. Dietary supplementation reduced the serum CHO and GPT concentrations across the goat groups (*P* < 0.05). Glucose concentration was higher in the TT group, followed by HM and CC, respectively (*P* < 0.05). Mean values of immunoglobulins, including IgG, IgA, and IgM were higher in goat group HM that received herbal mixture (P < 0.05).

**Table 2:** Effect of tannin (TT) and herbal mixture (HM) supplementation on serum biochemical parameters and immunological response in goats.

	Treatments						P-value
	CC		TT		HM		
	Mean	SE	Mean	SE	Mean	SE	
Glucose, mg/dl	49.08^a^	1.75	70.66^b^	6.62	56.36^a^	1.45	0.015
Cholesterol, mg/dl	88.02^b^	3.13	57.1^a^	7.68	75.09^b^	3.87	0.009
Triglycerides, mg/dl	46.43	3.57	48.57	10.69	52.85	5.34	0.83
Creatinine, mg/dl	0.97	0.1	1.24	0.084	1.09	0.1	0.19
Albumin, g/dl	4.08	0.16	3.63	0.21	3.852	0.1	0.22
Total Protein, g/dl	6.83	0.10	6.77	0.42	7.58	0.39	0.23
Globulins, g/dl	2.93	0.27	3.46	0.55	3.97	0.41	0.32
Urea, mg/dl	56.46	4.98	55.17	3.27	59.79	2.14	0.61
GPT, U/L	89.07^b^	6.49	82.85^b^	1.019	71.48^a^	1.52	0.011
GOT, U/L	15.75	1.25	16.8	2.62	15.2	1.46	0.83
IgG, mg/dl	50.98^b^	1.34	31.98^a^	0.53	78^c^	0.83	0.0001
IgA, mg/dl	31.81^b^	0.78	29.27^a^	0.33	35.40^c^	0.5	0.0001
IgM, mg/dl	15.61^b^	0.11	14.46^a^	0.33	17.2^c^	0.37	0.0001

^a,b,c^: Means in the same row with different superscripts differ significantly (*P* < 0.05).

### Carcass characteristics and meat quality

Table 3 shows the results of hot carcass weight, dressing percentage, the weight of carcass offals and wholesale cuts, traits of the best ribs (9–10–11), and meat quality. The results revealed that hot carcass weight and dressing percentages were similar between groups. The results of carcass offal weights revealed that the weight of renal fat declined due to the supplementation (*P* < 0.05) (Table 3). Similarly, the fat content of the best ribs was declined, and group TT showed the lowest fat content, followed by HM and CC, respectively (*P* < 0.05) (Table 4). Regarding meat quality parameters, yellowness was higher (*P* < 0.05) in the HM group, followed by CC and TT, respectively. Meat Hue was higher in group HM (*P* < 0.05) and lower in the TT group (Table 3).

**Table 3:** Carcass characteristics and meat quality of goats supplemented with tannin (TT) and herbal mixture (HM).

	Treatments						P-value
	CC		TT		HM		
	Mean	SE	Mean	SE	Mean	SE	
Slaughter Weight, kg	36.46	0.49	34.49	3.22	36.53	1.035	0.74
Hot Carcass, kg	19.05	0.22	17.99	1.72	18.81	0.56	0.79
Dressing, 1%	52.25	0.14	52.1	0.4	51.5	0.22	0.20
**Carcass offals**							
Heart, g	128.33	6.01	118.33	4.41	118.33	6.01	0.39
Liver, g	518.33	41.06	466.66	35.28	451.66	14.81	0.37
Kidneys, g	105	2.89	100	5.00	123.33	19.22	0.38
Spleen, g	46.66	3.33	56.66	10.14	51.66	4.41	0.59
Lungs & trachea, g	538.33	29.06	420	57.95	520	16.07	0.14
Renal fat, g	210^b^	30.55	83.33^a^	16.41	135^ab^	20.21	0.023
Abdominal fat, g	555	102.59	513.33	52.39	391.66	76.50	0.38
**Wholesales cuts**							
Head, kg	2.31	0.09	2.25	0.25	1.66	0.17	0.08
Pelt, kg	3	0.25	3	0.29	2.83	0.17	0.85
Feet, kg	1.10	0.10	0.81	0.20	1.00	0.03	0.33
Shoulders, kg	4.16	0.15	3.67	0.36	3.46	0.25	0.25
Racks, kg	5.04	0.31	4.78	0.34	4.41	0.34	0.44
Flank, kg	0.65	0.03	0.50	0.11	0.35	0.04	0.06
Lion, kg	1.00	0.12	0.96	0.09	0.90	0.09	0.78
Legs, kg	5.13	0.17	4.53	0.31	4.23	0.20	0.08
**Best ribs 9–10–11**							
Best ribs 9–10–11, kg	0.72	0.03	0.65	0.027	0.66	0.06	0.41
Lean, g	0.36	0.05	0.35	0.047	0.36	0.06	0.99
Bone, g	0.21	0.01	0.19	0.01	0.18	0.01	0.15
Fat, g	0.18^c^	0.01	0.08^a^	0.01	0.11^b^	0.00	0.001
**Meat quality**							
WHC	67.8	2.31	65.2	2.28	65.86	4.23	0.83
Brightness	43.54	1.48	43.79	1.87	45.13	1.75	0.78
Redness	18.73	0.99	16.34	0.39	16.10	0.68	0.08
Yellowness	9.40^a^	0.35	7.32^a^	0.30	10.03^b^	0.33	0.003
Chroma	20.96	1.02	17.91	0.31	18.98	0.58	0.056
Hue	26.69^a^	0.66	24.15^a^	1.22	31.98^b^	1.47	0.008

^a,b,c^: Means in the same row with different superscripts differ significantly (*P* < 0.05).

**Table 4:** Chemical composition and fatty acids profile in meat of goats supplemented with tannin (TT) and herbal mixture (HM).

	Treatments						P-value
	CC		TT		HM		
	Mean	SE	Mean	SE	Mean	SE	
**Chemical composition, g/kg**							
Moisture	717.80	5.7	731.80	10.80	720.30	12.20	0.59
Protein	196.10	3.2	200.60	1.10	201.20	1.30	0.24
Fat	53.6	2.2	35.2	10.10	47.7	10.60	0.36
Collagen	18.10	2.0	21.40	3.30	18.20	4.40	0.74
**Fatty acids profile, %**							
C14:0, (tertadecanoica)	1.98	0.14	1.10	0.45	1.56	0.30	0.23
C15:0, pentadecanoic	0.19	0.07	0.28	0.03	0.34	0.02	0.12
C16:0, Palmitic (hexadecanoic)	25.44^b^	0.37	25.90^b^	0.08	24.33^a^	0.29	0.02
C17:0, Heptadecanoic	0.80^a^	0.04	0.82^a^	0.05	1.05^b^	0.04	0.01
C18:0, Stearic (octadecanoic)	30.1	0.63	30.17	4.44	26.66	1.33	0.60
∑ SFA	58.53	0.36	58.29	4.79	53.96	0.89	0.48
C16:1 7cis, Palmitoleic (9-hexadecenoic)	0.19	0.09	0.11	0.01	0.19	0.02	0.46
C16:1 9 cis, Palmitoleic (9-hexadecenoic)	0.9^a^	0.04	0.76^a^	0.13	1.81^b^	0.15	0.001
C18:1 9cis, oleic (octadecenoic)	36.91	0.43	38.22	4.79	39.48	0.78	0.81
C18:1 13 Trans,	0.68^b^	0.02	0.73^b^	0.09	0.28^a^	0.00	0.002
C18:1 6 cis,	1.34	0.07	0.81	0.24	2.07	0.56	0.10
∑ MUFA	40.03	0.39	40.63	4.97	43.85	1.48	0.64
C18:2 −9–12 cis, linoleic (octadecadienoic)	1.25	0.03	0.78	0.02	1.41	0.30	0.098
C18:3—cis 6,9,12, α-Linolenic (6,9,12-octadecatrienoic)	0.14^a^	0.01	0.16^a^	0.03	0.44^b^	0.07	0.007
∑ PUFA	1.39^ab^	0.02	0.95^a^	0.03	1.85^b^	0.25	0.013

^a,b,c^: Means in the same row with different superscripts differ significantly (*P* < 0.05).

### Chemical composition and fatty acid profile in goats’ meat

Small numeric differences in meat moisture, protein, fat, and collagen were observed without significant differences (Table 4). Some of the fatty acids in goat meat were affected by dietary supplementation (Table 4). The concentration of Palmitic declined in the HM group compared to the CC and TT groups (*P* < 0.05). The supplementation increased Heptadecanoic, Palmitoleic, α-Linolenic, and polyunsaturated fatty acids (PUFA) (*P* < 0.05) in the HM group compared to the CC and TT groups (Table 4).

### Histological examination of muscle and intestine

**Fig. 1:**
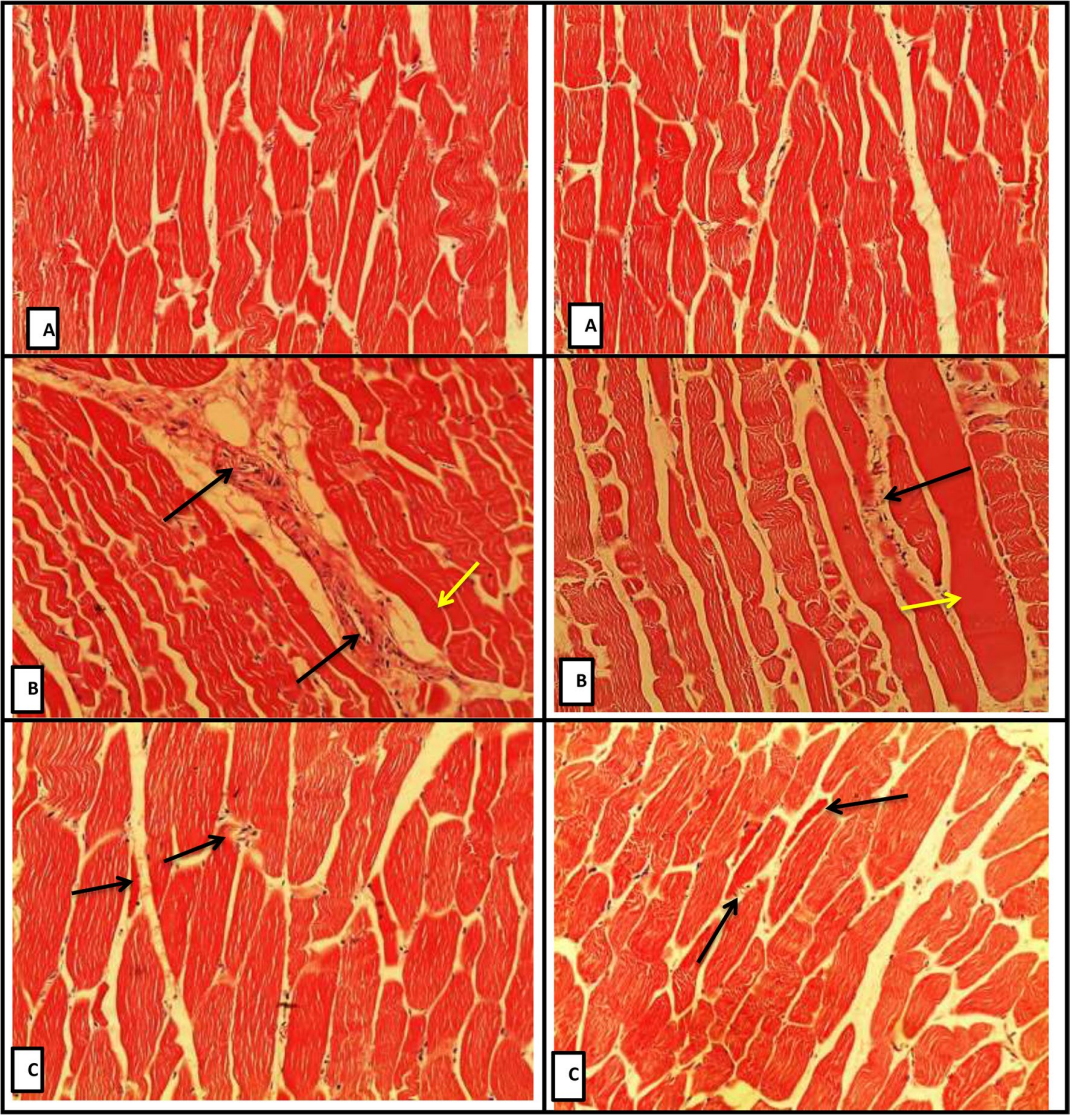
The histology of goat muscle as affected by tannin and herbal mixture compared with control group. (**A**) refers to normal appearance muscle of CC goats (H&E, X10); (**B**) showing zenker necrosis of muscle (yellow arrow), infiltration of inflammatory cells and myolysis (black arrows) in the muscles of TT goats (H&E, X10);(**C**) showing normal appearance muscle of goat with slight infiltration of inflammatory cells and slight necrosis (black arrows) in the muscles of HM goats’ group (H&E, X10).

**Fig. 2:**
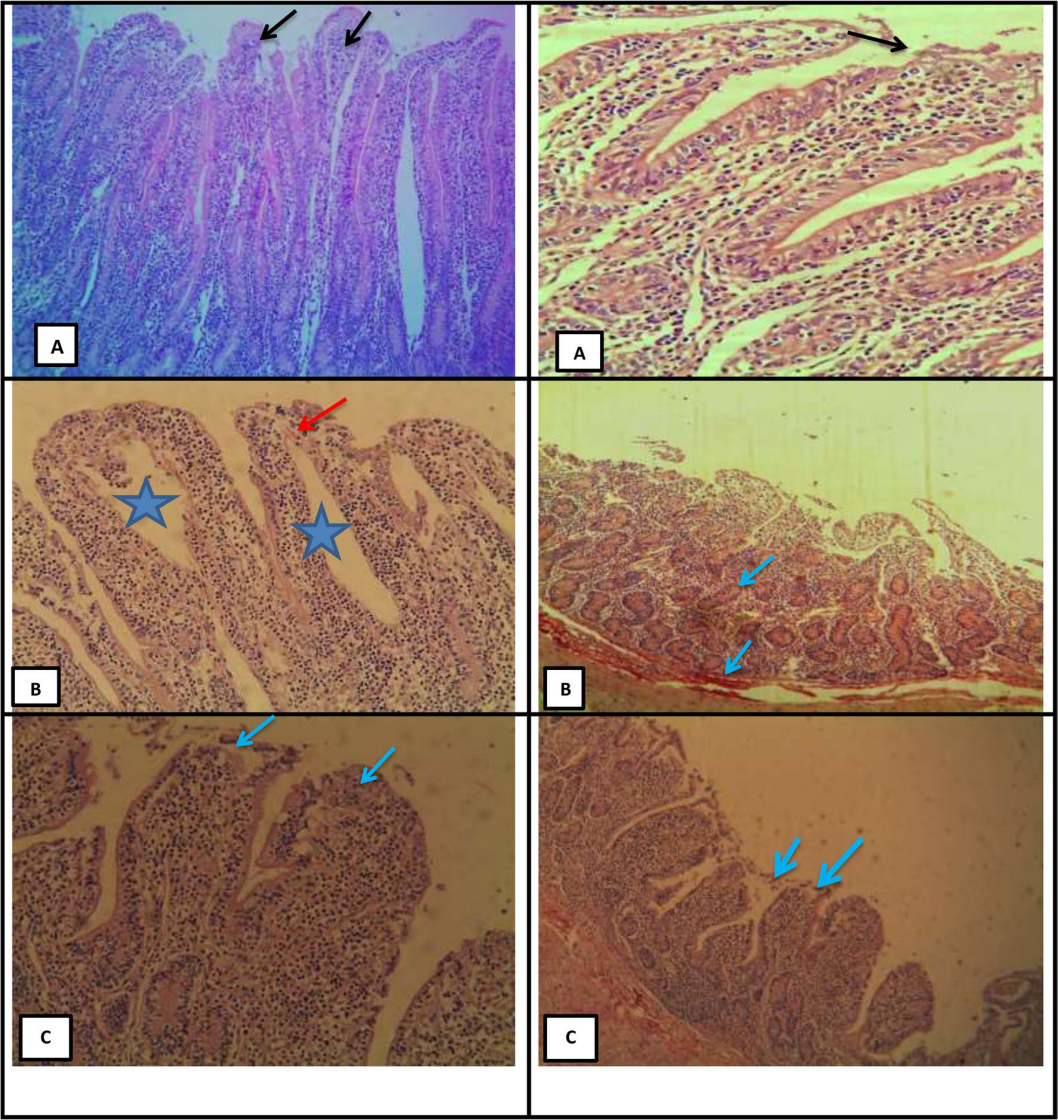
The histology of goat intestine as affected by tannin and herbal supplementation. (**A**) refers to intestine of CC goats and showing destructive changes in the epithelial lining of the intestinal villi (black arrow) (H&E, X10); (**B**) refers to intestine of TT goats and showing catarrhal enteritis, swelling of the villi and edema (star) besides clear destructive changes in the epithelial lining of the intestinal villi (red arrow); (**C**) refers to intestine of HM goats and showing destructive changes in the epithelial lining of the intestinal villi (black arrow), desquamated epithelial cells and infiltration of chronic inflammatory cells (red arrow).

Examination of muscle showed normal appearances of striated muscle in the control and HM groups, while muscle necrosis infiltration and slight myolysis were detected in group TT (Fig. 1). The histopathological examination of the intestine (Fig. 2) showed long villi with a slight degenerative change in the epithelial lining in group CC. While catarrhal enteritis and chronic enteritis can be detected in group TT with a clear appearance of swelling of villi and edema, severe infiltration of chronic inflammatory cells between intestinal glands, and congestion of submucosal blood vessels. Additionally, the intestine of group HM showed slight destructive changes in villi and desquamated epithelial cells within the infiltration of inflammatory cells.

## Discussion

### Animal performance

The herbal mixture that was used in this study contains phenols, tannins, flavonoids, and saponins and showed positive effects on growth performance, feed utilization, immunity, resistance against intestinal parasites, and meat quality, which agree with similar studies^1,6^. The effect of current dietary treatments on the rumen fermentation parameters and rumen bacteria and archaea were described previously in Rabee et al.^14^.

In the present study, the ADG showed an increment due to the supplementation (Table 1), which agrees with previous studies on herbal mixtures and tannins^1,6,7^. The difference in growth rate was not significant between the CC and TT groups, which agrees with the findings of goat supplemented with tannin-rich ground pine bark^28^. The improvement in the growth performance in HM was accompanied by an improvement in the digestibility of nutrients (Table 1), which agrees with the findings of sheep supplemented with a herbal mixture^1^. In addition, garlic supplementation increases the synthesis of vitamin B6, which improves the digestibility and growth performance of animals^29^. Similarly, the digestibility was higher in lambs supplemented with saponin, which is an essential component in the herbal mixture^7^. In another study, tannin and herbal mixture supplementation improved the production of volatile fatty acids (VFA), which increases the energy supply to animals^14^. Furthermore, the improvement in feed utilization can be attributed to the declines in rumen methanogens and methane emission as well as the enhanced activity of rumen cellulolytic bacteria such as *Prevotella* and *Rikenellaceae RC9 gut group* due to the phytogenic supplementation^14,30,31^. Methane emission represents a loss in energy feed intake up to 12%^14^. Higher nitrogen balance in the HM group could indicate lower nitrogen excretion^32^, and higher incorporation of nitrogen into microbial protein synthesis as a result of stimulated rumen microbial activity as a result of phytochemical supplementation^33,34^. Moreover, the inclusion of condensed tannin in group TT did not negatively affect the digestibility of CP and EE and improved the digestibility of DM, OM, and NDF (Table 1). The concentration of condensed tannin in the current study was 1%, which explains the positive effect of tannin compared to the control group. Previous research has suggested that 2–4% CT based on DM is suitable to maintain favorable growth performance and rumen fermentation^9^.

### Fecal parasites and blood metabolites and immunity

Giardia and Strongyloides parasites are commonly observed in small ruminants, and infected animals showed a reduction in growth and poor health^35^. Phytogenic compounds have anti-parasite activity^36^, which supports the decline of Strongyloides and Giardia in TT and HM groups in the present study. Similarly, supplementation with acacia, which is rich in tannin, decreased fecal egg count in goats^37^. Moreover, garlic supplementation decreased the parasite count and improved the growth performance in sheep and goat^36,38^. Phytogenic feed additives reduce the production of parasites’ eggs by reducing the fecundity of adult worms and improving the immune response^28,39^. Additionally, sulfur compounds of herbal plants control the parasites by impacting their physiological processes^40^. Furthermore, phytochemicals boost the immunity by increasing the production of blood immunoglobulins^1,34^, which supports the higher serum IgA, IgG, and IgM in group HM. Furthermore, the decline in parasite count in the host animal improves the immune response^28,39^. Additionally, phytogenic compounds stimulate rumen fiber-degrading bacteria that boost the animal’s immunity by representing a barrier against pathogens^41^.

The herbal mixture supplementation improved blood glucose and decreased blood cholesterol (Table 2), which agrees with studies that applied phytogenic supplementation^1,39^. The improvement in blood glucose is attributed to higher carbohydrates (NDF) digestibility^7,28^. Recent findings have shown that garlic contains organosulfur compounds that inhibit cholesterol biosynthesis^3^, which explains the current findings. The decline in serum GPT refers to the fact that supplementation had no harmful side effects on the liver^28^. Thus, the improved growth performance could be attributed to the modulation in the rumen microbial ecosystem, which resulted in improved feed utilization, nitrogen balance, VFA supply, and enhanced immunity as well as declined parasite counts and methane production.

### Carcass characteristics and meat quality

Tannin and herbal mixture supplementation did not negatively affect carcass characteristics or meat quality negatively, which agrees with findings of previous studies on goats supplemented^28,42^. The increase in the meat yellowness in group HM could be attributed to the pigment in the herbal mixture that could change the meat color^43^.

Tannin and herbal mixture supplementation decreased the renal fat and fat proportion in meat, which is consistent with previous studies on sheep and goats^1,13,28^. In the same line, sheep supplemented with *Astragalus membranaceus* roots, that is rich in saponins, polysaccharides, and flavonoids, showed lower meat fat compared to the control group, which supports our findings^44^. Furthermore, lower meat fat could be a result of the decline in fatty acid synthesis and the increase in fatty acid β-oxidation due to phytogenic supplementation^45,46^. Fatty acids content, especially the unsaturated fatty acids, have a substantial effect on the quality of meat. The decline in palmitic acid in the HM group is a positive point of herbal mixture supplementation, as palmitic acid is harmful to serum cholesterol levels in humans^13^. In this study, some unsaturated fatty acids were improved due to herbal supplementation. Chanjula et al.^13^ indicated that phytochemical supplementation is an effective technique to increase beneficial polyunsaturated fatty acids (PUFA) in red meat, which highlights the higher PUFA in the HM group as polyunsaturated fatty acids (PUFA) provide several health benefits for the consumers.

### Histological examination

Histological analysis of muscle and intestine (Figs. 1 and 2) indicated that tannin supplementation influenced the tissues negatively. Previous results reported that phytochemicals caused necrosis in the histology of ruminant meat^47,48^. Acacia hay that is rich in tannin caused negative histological changes on the small intestine of sheep, including a focal inflammatory cells infiltration and edemas in the lamina propria of the mucosal layer^49^. The negative effect of tannin on the intestine could be a result of excessive production of mucus, the shift in rumen VFA, and the decline in cell turnover^14,50^. Tannins decrease the availability of nutrients by affecting protein cross-linking and mineral chelation, which causes a decline in villus height and crypt depth^51^. No clear explanation of the negative effect of tannin on the tissue morphology in animals. However, Buyse et al.^51^ reported that tannins and their metabolites can be cytotoxic and inhibit cell proliferation.

This study provided a piece of new knowledge about the effect of phytochemicals on animal performance and health. However, some barriers constrained the generation of a more comprehensive study, such as the availability of animals to compare more extracted and separated phytochemicals to the herbal mixture. Though the non-significance of the size effect and power analyses, more animals in each experimental group are recommended (> 9).

In conclusion, the utilization of herbal supplementation appears to exhibit a positive effect on feed utilization, blood serum immunity, and decreased fecal parasites, which resulted in improved growth performance. Additionally, the tannin and herbal mixture supplementation decreased carcass renal fat and abdominal fat as well as meat fat and increased polyunsaturated fatty acids, which enhance the meat quality and provide health benefits to humans.

## Supplementary Information

Supplementary Information.

## Data Availability

The raw data of this study is available by the corresponding author up on request.
