# Supplementary material for: Growth performance, carcass traits, and histological changes of goats supplemented with different sources of phytochemicals
Source: Sci Rep. 2025 Jun 23;15:20264. doi: 10.1038/s41598-025-07306-w (PMC12185740; doi:10.1038/s41598-025-07306-w)
Supplement: Supplementary file 1 — Supplementary Information. [file 41598_2025_7306_MOESM1_ESM.docx]

| **Name of device, kits, or tool** | **The origin** |
| --- | --- |
| Meat analyzer | FOSS, Denmark |
| Chroma meter | Konica Minolta, model CR 410, Japan |
| Blood metabolites commercial kits | Spectrum Biotechnology, Egypt |
| Gas chromatography (GC) trac 1300 | Thermo Fisher Scientific, Waltham, MA USA |
